# Neuro-Transistor Based on UV-Treated Charge Trapping in MoTe_2_ for Artificial Synaptic Features

**DOI:** 10.3390/nano10122326

**Published:** 2020-11-24

**Authors:** Shania Rehman, Muhammad Farooq Khan, Mehr Khalid Rahmani, Honggyun Kim, Harshada Patil, Sobia Ali Khan, Moon Hee Kang, Deok-kee Kim

**Affiliations:** 1 Department of Electrical Engineering, Sejong University, 209 Neungdong-ro, Gwangjin-gu, Seoul 05006, Korea; shania.rehman19@gmail.com (S.R.); khgking11@naver.com (H.K.); Harshadapatil.nanotech@gmail.com (H.P.); 2 School of electronics Engineering, Chungbuk National University, Cheongju 28644, Korea; Mehrkhalid.2nd@gmail.com (M.K.R.); Sobiaali717@gmail.com (S.A.K.); moonhee@chungbuk.ac.kr (M.H.K.); 3 Department of Convergence Engineering for Intelligent Drone, Sejong University, Seoul 05006, Korea

**Keywords:** MoTe_2_ transistor, chemical synapse, neuromorphic, charge trapping and artificial synaptic

## Abstract

The diversity of brain functions depend on the release of neurotransmitters in chemical synapses. The back gated three terminal field effect transistors (FETs) are auspicious candidates for the emulation of biological functions to recognize the proficient neuromorphic computing systems. In order to encourage the hysteresis loops, we treated the bottom side of MoTe_2_ flake with deep ultraviolet light in ambient conditions. Here, we modulate the short-term and long-term memory effects due to the trapping and de-trapping of electron events in few layers of a MoTe_2_ transistor. However, MoTe_2_ FETs are investigated to reveal the time constants of electron trapping/de-trapping while applying the gate-voltage pulses. Our devices exploit the hysteresis effect in the transfer curves of MoTe_2_ FETs to explore the excitatory/inhibitory post-synaptic currents (EPSC/IPSC), long-term potentiation (LTP), long-term depression (LTD), spike timing/amplitude-dependent plasticity (STDP/SADP), and paired pulse facilitation (PPF). Further, the time constants for potentiation and depression is found to be 0.6 and 0.9 s, respectively which seems plausible for biological synapses. In addition, the change of synaptic weight in MoTe_2_ conductance is found to be 41% at negative gate pulse and 38% for positive gate pulse, respectively. Our findings can provide an essential role in the advancement of smart neuromorphic electronics.

## 1. Introduction

Various attempts have been made to emulate the key functions of biological neurons in artificial intelligence and neuromorphic electronic devices for the next generation [1,2,3,4]. In a complementary metal–oxide–semiconductor (MOS) transistor, the von-Neumann architecture is insufficient to control the conventional constraints of computational difficulty and associated power consumptions [5]. Meanwhile, recent neuromorphic technology faces the challenges of high circuitry complexity, low efficiency, and difficulties of low-dimensional size of devices. However, recent developments in imaging techniques have led to the discovery of several significant and important details about information processing inside the human nervous systems which is precisely considered in electronic devices to develop more genuine and proficient human brain-inspired machine interfaces [6]. The scientists and physicians are encouraged to diagnose and treat various brain diseases and disorders by using these artificial machines. The main purpose of neuromorphic engineering and the human-brain scheme is to realize the function of brain which manages billions (~10^11^) of information processing units connected through trillions (~10^15^) of synapses. Neurons and synapses are basic units of brain memory and information processing which emulate neuromorphic responses via nano-electronic devices to achieve the objectives of machine learning, robust computational development, and short-term and long-term plasticity. It can also be helpful to design the artificial sensory systems which demonstrate the growth and advancement of diseases and disorders and likely effects of drugs, and autonomous and intelligent robots that will ultimately simplify difficult surgical techniques. To emulate the analog computing arrangement of one synapse, at least 10 complementary MOS transistors are justified [7]. Considering these architectures demand strongly interlinked elements, the scalability of these systems becomes a primary challenge [8]. Furthermore, compacting with the aforementioned issue, a large amount of power (>1000 W) is utilized by such platforms to execute tasks that the human brain completes with <4 W [9]. Therefore, this emphasizes the urgent demand to reveal the electronic devices that can mimic the synaptic or neuronal response which are attuned with the human brain. It is evident that the information collected through different human body parts/organs transform into electrical impulses. Later, these impulses are coped and processed by release of neurotransmitters inside the human brain, an intricate network of neurons [10]. Thereafter, the decisive results of the neural information processing is categorized into sort-term action (logic operation) and long-term changes (memory storage) [11]. Intentionally, it can be associated with two different mechanisms having different time constants in order to mimic both the short-term and long-term plasticity of the human brain [12,13]. However, the time constant for logic operation is principally controlled by the transit time of charge carriers (electrons/holes) from the source to the drain of FETs. The transit time of carriers is critical to determine the memory retention which mainly depends upon the conductance of channel material (MoTe_2_), charging and dis-charging of carriers and gate oxide. Several techniques have been adopted to mimic the neuromorphic transmission and human behavior in artificial devices. As such, transistors, memristors, and other devices are being used for this purpose and bi-state spin valve junctions could be the best choice for computing purposes [14,15,16,17,18,19]. Although the two-terminal devices have commendably demonstrated the synaptic systems with a facile rout of fabrication, they face some issues when performing weight training and signal transmission [20,21,22].

To account for these issues, the three-terminal devices like FETs have been introduced to mimic the synapse called “synaptic transistor”. Numerous oxide materials, carbon nanotubes, nanoparticles, and ferroelectrics have been investigated to emulate the neural electronics [23,24,25,26]. Recently, promising two-dimensional (2D) transition metal dichalcogenides (TMDCs) have shown a huge potential to be utilized in synaptic transistors and in a recent study a 2D-TMDCs disulphide group (MoS_2_ and ReS_2_) was adopted for neuromorphic computing [27,28]. However, less attention has been paid to the telliride (Te) group of 2D-TMDCs. Generally, for neuromorphic transistors researchers use special high-k dielectrics (HfO_2_, Al_2_O_3_ and TaO_x_) substrates for charge trapping purpose. However, we used simple Si/SiO_2_ substrates and subsequently the 2D material (MoTe_2_) is treated with UV in air to enhance the trapping mechanism. Our technique is proposed to be easy and novel for electron trapping in neuro-transistors.

## 2. Materials and Methods

First, we made large patterns on p^++^-Si/SiO_2_ substrates by photolithography and deposited Cr/Au (5/30 nm) by thermal evaporation to obtain big patterns. Initially, MoTe_2_ flaks were exfoliated in on scotch tape and transferred on polydimethylsiloxane (PDMS) stamp. Later, on PDMS MoTe_2_ was treated with DUV for 10 sec in ambient condition to encourage trapping states and finally transferred on SiO_2_ substrates with the help of a micromanipulator. At this moment, the treated side of MoTe_2_ was coincided with SiO_2_. Subsequently, using electron beam lithography, EL-9 and PMMA were spin-coated on the devices to make fine source and drain terminals. Lastly, Cr/Au, 7/80 nm, were deposited in thermal evaporation chamber and the lift-off was completed in acetone/methanol. Electrical measurements were performed by using Keithley-2400 (Keithley Instruments Inc., Solon, OH, USA) and Picoammeter Keithley-6485 (Keithley Instruments Inc., Solon, OH, USA). The pulsed measurements were performed using the Keithley-4200 (Keithley Instruments Inc., Solon, OH, USA) semiconductor characterization system (SCS) with additional pulse measurement units (PMU). The Raman spectrum was acquired using a Renishaw microspectrometer (Renishaw plc., Wotton-under-Edge, UK) with a laser excitation wavelength of 514 nm. Moreover, DUV light of 220 nm was used with an optical power of 11 mW cm^−2^.

## 3. Results and Discussion

**Figure 1:**
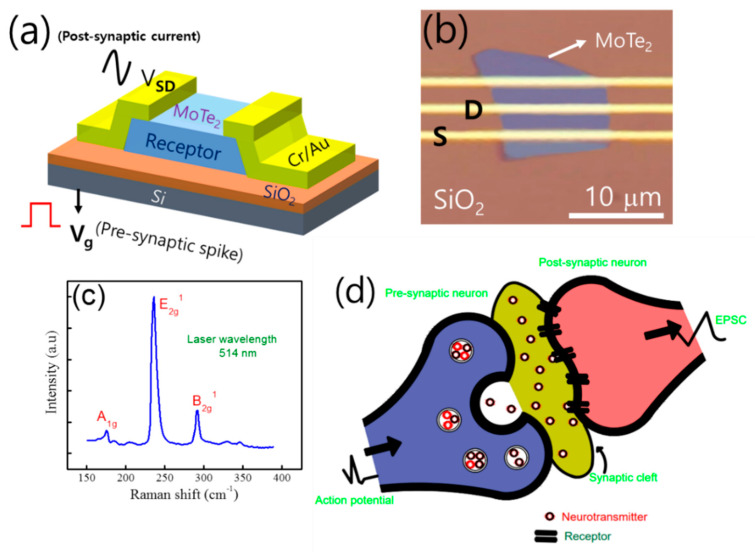
(**a**) The schematic illustration of MoTe_2_ three terminal field effect transistors (FET) where charge trapping and de-trapping dynamics at atomically thin channel 2D material/SiO_2_ interface. (**b**) The final optical microscope image of MoTe_2_ FET with Cr/Au electrodes. (**c**) Raman spectroscopy of few layer MoTe_2_. (**d**) Presentation of a biological synapse where action potentials control the release and absorption of neurotransmitters.

The schematic representation and final optical microscope image of MoTe_2_ field effect transistor (FET) are demonstrated in Figure 1a,b, respectively. The MoTe_2_ transistor is supposed to replicate the neuro-biological dynamics in a chemical synapse. In Figure 1b, the MoTe_2_ flake is transferred on a SiO_2_ substrate and the Cr/Au electrodes are deposited by thermal evaporation. Further detailed information is given in the experimental section. Raman spectroscopy is the best tool to characterize the band and crystal structure of materials. In Figure 1c, we showed the Raman spectrum of MoTe_2_ which demonstrated the few frequency modes at different positions. We characterized Raman spectroscopy MoTe_2_ with laser light having wavelength of 514 nm. The power intensity of laser light is kept at 1 mW/cm^2^. Thereby, three modes are observed, namely, A_1g_, E_2g_^1^ and B_2g_^1^ at ~174.6 cm^−1^, ~235.9 cm^−1^, and ~291.2 cm^−1^, respectively. Thus, our results are almost consistent with previous reports and confirmed that MoTe_2_ flake is multilayer in thickness [29]. For further confirmation we performed the atomic force microscopy (AFM) and investigate the exact thickness (~15 nm) of MoTe_2_ flake. The AFM image and height profile of the flake is shown in Figure S1a,b. The synapses have a fundamental role in the propagation of information in the central nervous system. In this type of structures, plasma membrane of the presynaptic neuron (signal-carrying neuron) comes very close to the plasma membrane of the postsynaptic neuron (target neuron). The schematic presentation of synaptic response where release and absorption of neurotransmitter are controlled by action potentials is shown in Figure 1d.

**Figure 2:**
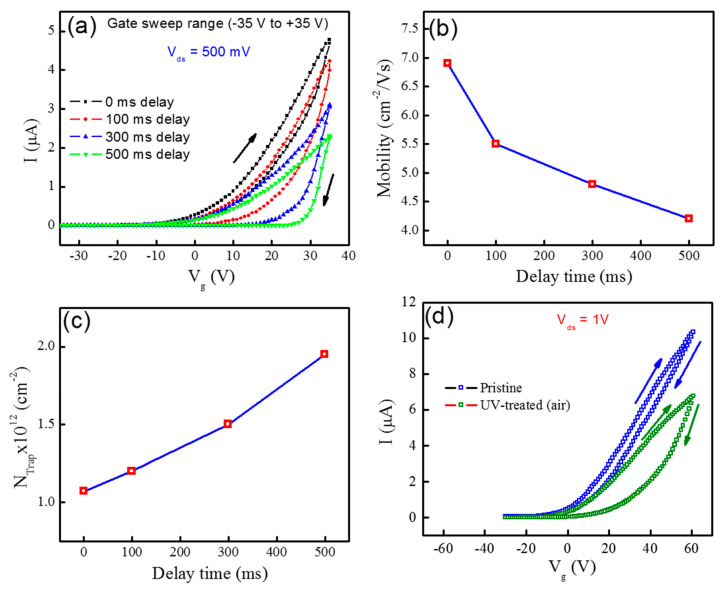
(**a**) Transfer characteristics of the MoTe_2_ FET having hysteresis loops at different delay times. (**b**) Mobility as a function of the delay time of MoTe_2_ FET. (**c**) Delay time dependent effective trap states density of MoTe_2_/SiO_2_ FET. (**d**) The transfer curves before and after UV treatment (10 s) of MoTe_2_/SiO_2_ FET.

We performed our all electrical measurements in ambient conditions where the drain current acts as the EPSC, which is supposed to be the device conductance as a function of time. Moreover, the pre- and post-synaptic pulses have been applied to the back-gate terminal (p-Si/SiO_2_) and the MoTe_2_ channel with the trapped states behaves as an analog to the synaptic fluid where neurotransmitters are released and absorbed. However, in Figure 2a we measured the transfer characteristics of MoTe_2_ transistor in forward and revers sweep from −35 V to +35 V and then from +35 V to −35 V. Here, we found n-type semiconductor by fixing V_ds_ = 500 mV owing gate voltage gap size near 1 V. It is evident from Figure 2a that we observed a promising hysteresis loop in transfer characteristics at 0 ms holding/delay time of the gate sweep, which is commonly attributed to the donor like trap/defect states formed at MoTe_2_/SiO_2_ interface and also due to top surface adsorbents/water dipoles on MoTe_2_ [30,31]. Also, as we increased the holding/delay time in transfer characteristics the size of hysteresis loop becomes large, as shown in Figure 2a. It is noted in a previous report that at short delay time, the charges are trapped only in shallow levels and the hysteresis is found to be minor. However, after increasing the delay time, the charges start being trapped in deep levels due to which a large number of electrons are trapped in both the shallow and deep levels. Therefore, the hysteresis loop become significant at large delay time [32]. We proposed that by increasing the delay time, we may improve the effect of trapping states on charge carries. Therefore, to see in depth we calculate the mobility of MoTe_2_ transistor at different holding/delay times. The mobility of MoTe_2_ device is obtained using the following Equation (1):

(1) $$\mu =\frac{L}{{\mathrm{WC}}_{g}V_{\mathrm{ds}}}\frac{{\mathrm{dI}}_{\mathrm{ds}}}{{\mathrm{dV}}_{g}}$$

where ${\mathrm{dI}}_{\mathrm{ds}}/{\mathrm{dV}}_{g}$ is the slope of the transfer curve in the linear region, C_g_ is the gate capacitance (~115 aF μm^−2^) of Si/SiO_2_ substrates V_ds_ is the drain voltage, L is the channel length, and W is the width. In Figure 2b, the mobility is decreased from (6.9, 5.5, 4.8 and 4.2 cm^−2^/Vs) as the delay time of gate sweep is increased from (0, 100, 300, and 500 ms) which endorsed the enhanced influence of trapping states with increased delay time. To estimate the equivalent trap density (NTrap) at the MoTe_2_/SiO_2_ interface the following Equation (2) is used:

N(Trap) = C_g_[V_th_(Forward) − V_th_(Reverse)]/q (2)

where C_g_ = ~115 aF μm^−2^ is the gate capacitance of back gate, V_th_ is the threshold voltage (Forward and Reverse) and q is the electric charge. The N_Trap_ of n-FET based MoTe_2_ is 1.07 × 10^12^, 1.2 × 10^12^, 1.5 × 10^12^ and 1.95 × 10^12^ cm^−2^ for 0, 100, 300, and 500 ms holding time by gate weep as shown in Figure 2c. To get additional understanding into the charge trapping mechanism, the hysteresis loops of the transistor are also measured at different back gate sweeping ranges (−35 V to +35 V, −25 V to +25 V and −15 V to +15 V) as shown in Figure S2a (Supplementary Information). The variation in the hysteresis loops at different sweep rates designates the existence of slow charge trapping in defect states. The observed change in I_ON_ of the transistor is usually due to the random trapping and de-trapping of the charge carriers in the MoTe_2_ or SiO_2_. Also, we demonstrated the effect of UV on the bottom side of MoTe_2_ flake by measuring the transfer curves of FET. We can see in Figure 2d that the hysteresis loop becomes large compared to the pristine one. In the pristine device the hysteresis loop is ΔV = ~5 V but after UV treatment the loop is improved to ΔV = ~15 V.

**Figure 3:**
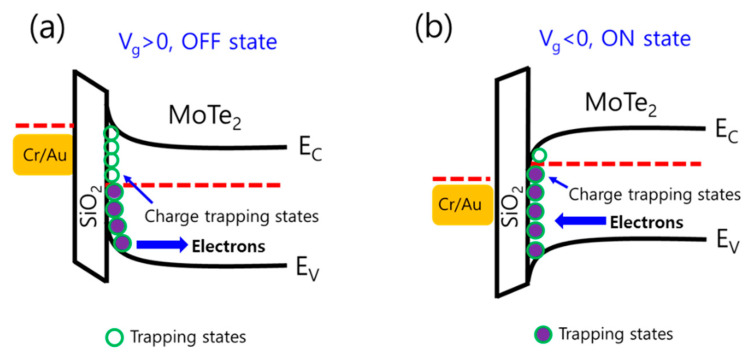
Schematic band diagram of MoTe_2_ FET with trapped states at the MoTe_2_/SiO_2_ interface. (**a**) When negative gate is applied. (**b**) When positive gate is applied.

To explain further, the trapping and de-trapping of the charge carriers are demonstrated by the energy band diagram as shown in Figure 3. According to Figure 3a when the applied back gate voltage is negative (V_g_ > 0, off state), the induced electric field in SiO_2_ (dielectric) will encourage the trapped electrons to be released from trapped states into the n-semiconductor MoTe_2_ channel and produce a large current density, and as a result the conductivity of MoTe_2,_ will be enhanced. On the other hand, in Figure 3b, if we applied positive pulse of back gate voltage (V_g_ < 0, on state), then the induced electrical field will deplete the electrons from the MoTe_2_, thereby reducing the channel conductivity of MoTe_2_. This situation give rise to the shift in threshold voltage of the transistor, which is consistent with the previous report [28]. The variation of channel conductivity can be interlinked with the synaptic plasticity of neurons. Our work is mainly based on the trapping and de-trapping of charge carriers in MoTe_2_ FETs where we need to examine their behavior to emulate the human synaptic functions and learning rules like LTP/LTD and various learning mechanisms such as SADP and STDP. These activities of neuromorphic function are based on different single, double, and successive positive and negative back gat pulses. Therefore, the back gate behaves like the pre-synaptic terminal and controls the conductance of the MoTe_2_, which acts like a synapse.

**Figure 4:**
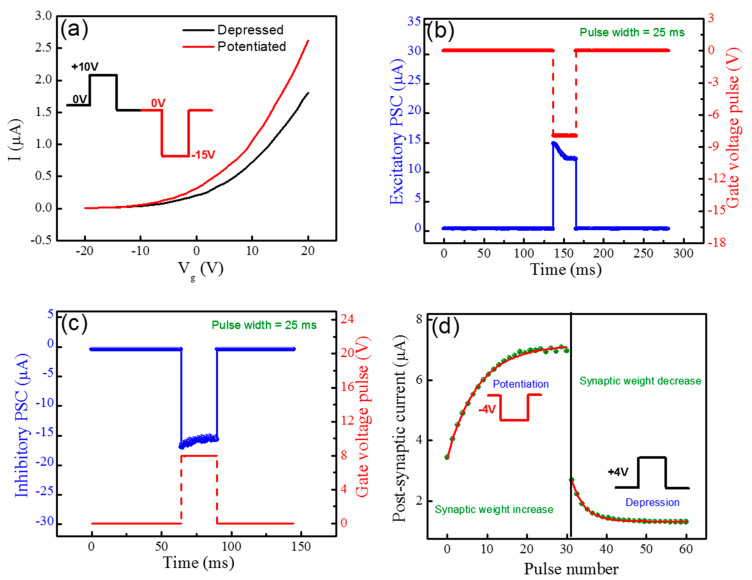
(**a**) Change in the threshold voltage of MoTe_2_ FET after 50 consecutive pulses to the gate terminal. (**b**) The excitatory post-synaptic current (EPSC) changes in MoTe_2_ synaptic transistor at negative gate pulse. (**c**) The inhibitory post-synaptic current (IPSC) changes in the MoTe_2_ synaptic transistor at positive gate pulse. (**d**) The application of negative and positive voltage pulses (V = −4 V and +4 V) demonstrating exponential trend in PSC, showing long-term depression (LTD) and long-term potentiation (LTP) processes, respectively.

Subsequently, the drain acts as the post-synaptic terminal. Hence, the hysteresis effect in the transfer characteristics of the MoTe_2_ transistors is used to mimic the vibrant plasticity. Also, we can control the gate pulses to trap and de-trap the electrons from the MoTe_2_ and modify the channel conductance to describe the EPSC. As given in Figure 4a, a significant change is observed in the threshold voltage of MoTe_2_ transistor before and after applying a train of 50 pulses to the back-gate terminal. The amplitude and width of the pulse are −15 V and 300 ms, respectively, for potentiation (red). In addition, to recover the initial position of the threshold voltage, a train of 50 pulses are applied with same width and amplitude in opposite polarity +10 V to get a depression (black). However, this kind of measurement, when the pulse is applied to the gate terminal, can control the different repeated and reversible conductance states of MoTe_2_. Moreover, when a single voltage pulse (single spike response) is given the synapse can be either excitatory (negative gate pulse) or inhibitory (positive gate pulse) in nature. Nevertheless, it depends upon the tendency of increments in the post-synaptic current to threshold or decrement in the post-synaptic current away from the threshold. For only single gate voltage pulse, we give the height of gate pulse is −8 V by keeping the pulse width of 25 ms at 500 mV bias voltage. At this moment, the EPSC current reaches up to ~15 μA and then gradually comes back to the original position after the pulse ends, as shown in Figure 4b. The increase of EPSC current in MoTe_2_ channel is due to the release of trapped electrons into the MoTe_2_ channel from trapping states in the SiO_2_/MoTe_2_ interface. Here, the amplitude of the PSC triggered by the presynaptic spike is related to the synaptic strength/weight in the biological synapse. Similarly, when a positive gate voltage pulse is applied with height pulse = +8 V reduces the IPSC current reduced to ~−17 μA, attributed to the injected electrons from MoTe_2_ channel to the SiO_2_/MoTe_2_ interface which can emulate the IPSC behavior of biological synapse, as shown in Figure 4c.

**Figure 5:**
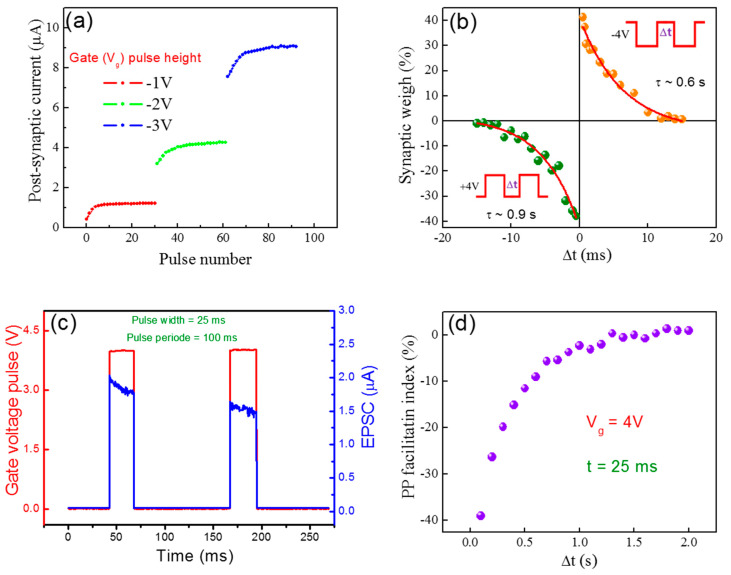
(**a**) Post-synaptic current vs. pulse number at different gate voltage pulse height, mimicking spiking amplitude-dependent plasticity. (**b**) The change in synaptic weight as a function of the time interval (Δt) between paired gate pulses of +4 and −4 V. (**c**) The change in EPSC after the application of two closely spaced pulses are applied to the gate terminal. (**d**) Quantification of STP using pulse paired facilitation measurements. Paired pulse facilitation (PPF) index designates the % change in the PSC due to the second pulse.

The merit features of synaptic plasticity are that of long-term potentiation (LTP) and long-term depression (LTD). These features can play essential role in the information processing of the biological nervous system [33,34]. In neuro-electronic synapse, LTP and LTD can be mimicked by using multiple pulses (spikes) of high frequency and in this way the synaptic transmission can be increased or decreased, respectively as shown in Figure S2b,c. Here, a train of 80 gate voltage pulses are given to the gate as pre-synaptic input for the investigation of drain current (post- synaptic current). It is found that a positive gate pulse heights (+5, +10 and +15 V) and a negative gate pulse height (−5, −10 and −15 V) generate negative and positive exponential changes in post-synaptic current, respectively, which indicate the LTP and LTD actions of a synapse. Further, the time constants for potentiation (at V_g_ = −4 V) and depression (at V_g_ = +4 V) responses are calculated from the experimental data which is found to be 2.9 and 2.7 s, respectively, as shown in Figure 4d. The conductance variation of MoTe_2_ channel is asymmetric with sudden increase and decrease in early potentiation and depression spikes and then it became saturated. We have also investigated the post-synaptic current by applying the different heights of gate voltage pulses, thereby the post-synaptic current experiences an increase/decrease in the channel conductance of MoTe_2_ with an increase/decrease in the gate height pulse, respectively, as shown in Figure 5a. Figure 5a describes the emulation of spike amplitude-dependent (SADP) of the biological synapse, since when the height applied gate voltage increases, more trapping states become activated and start to trap more electrons at the interface. The STDP in neuromorphic systems is another important function for the learning and memory process of a human brain. Thus, by increasing the time between two consecutive action potentials of pre-synaptic and post-synaptic spikes, the synaptic weight strength between two neurons can be changed [35]. Therefore, in our work the transistors mimicked the indirect STDP with a 41% change in MoTe_2_ channel conductance for negative gate pulse (V_g_ = −4 V) and a 38% change for positive gate pulse (V_g_ = +4 V), as shown in Figure 5b. Hence, this change is attained by paired gate pulses with a specific time period interval [36]. It is also important to note that as Δt increases, the change in channel conductance of MoTe_2_ become saturated because trapped electrons take a lot of time to be de-trapped. We studied the time constants for potentiation and depression pulses, which are 0.6 and 0.9 s, respectively, which seems plausible with the response times of biological synapses [35]. The time constant of relaxation for the potentiation and depression pulses are estimated by an exponential fit to the STDP data by using the following Equation (3):

(3) $$\Delta W\alpha \{\begin{array}{c} \exp\left(-\frac{\Delta t}{\tau_{+}}\right),\;\mathrm{if}\;\Delta t\geq 0\\ -\exp\left(\frac{\Delta t}{\tau_{-}}\right),\;\mathrm{if}\;\Delta t\leq 0 \end{array}$$

Furthermore, we observed the effect of double spike response of the MoTe_2_ synaptic transistor through the application of pair pre-synaptic pulses on the gate terminal. The paired pulsed facilitation/depression (PPF/PPD) is analog short-term synaptic plasticity, which is important to elaborate the time-based information in auditory or visual signals [37].

Therefore, EPSC or IPSC response can be observed at drain when two consecutive pre-synaptic spikes are applied to the gate terminal. As such, the enhancement or depression of PSC will occur because the back diffusion of the electron is not completed before the arrival of the second gate pulse. Thus, the post-synaptic current due to second pulse is lower than the first gate pulse. Substantially, as time increases the change in MoTe_2_ conductance is reduced due to the second pulse A_2_ or becomes equal to the first pulse A_1_ for higher time interval as shown in Figure 5c. Hereafter, we describe the PPF index as:

[(A_2_ − A_1_)/A1] × 100(%) (4)

Here, we have plotted the PPF index (%) versus interval from a positive gate voltage (V_g_ = 4 V) and pulse width = 25 ms) as shown in Figure 5d. Therefore, it is found that as we increased the time interval (Δt) between the pair of applied gate pulses the A_2_/A_1_ (PPF ratio) decreased. It is fitted by the exponential relation:

(5) $$\mathrm{PPF}=-1-A_{1}e^{\frac{\Delta t}{\tau 1}}$$

where A_1_ is the initial facilitation value and τ_1_ is the time relaxation of the phase. The calculation of the plasticity, PPF and STDP related time constants of relaxation can be used to initiate circuit level simulations for spiking neural networks. The ability of electrons trapping/de-trapping at interface can open a new avenues in future neuromorphic applications.

## 4. Conclusions

In summary, we demonstrated the tailoring of hysteresis loops in n-type MoTe_2_ FETs to emulate the biological synapses. We investigated that by modulating the amplitude and polarity of gate voltage pulses, we can precisely capture the excitatory or inhibitory nature of neurotransmitter. The trapping and de-trapping of electron from trapping states, formed at SiO_2_/MoTe_2_ interface, exhibit relaxation time constants and allow for the emulation of both STP and LTP which is an essential characteristic of learning and memory in biological synapses. In addition, we discussed the STDP, PFF, potentiation, and depression to replicate the human brain for advanced neuromorphic computing. The use of 2D MoTe_2_ as channel material on SiO_2_ substrate is a next-generation semiconducting material which offers outstanding device dimensions down to a single atomic thickness with diverse synaptic applications.

## Supplementary Materials

Supplementary materials can be found at https://www.mdpi.com/2079-4991/10/12/2326/s1.
