# Supplementary material for: Neuro-Transistor Based on UV-Treated Charge Trapping in MoTe2 for Artificial Synaptic Features
Source: Nanomaterials (Basel). 2020 Nov 24;10(12):2326. doi: 10.3390/nano10122326 (PMC7761516; doi:10.3390/nano10122326)
Supplement: Supplementary file 1 [file nanomaterials-10-02326-s001.pdf]

Supplementary Information

Neuro-Transistor Based on UV-Treated Charge Trapping in $MoTe_2$ for Artificial Synaptic Features

Shania Rehman [1,†], Muhammad Farooq Khan [1,†,*], Mehr Khalid Rahmani [2], Honggyun Kim [1], Harshada Patil [1,3] Sobia Ali Khan [2], Moon Hee Kang [2] and Deok-kee Kim [1,3*]

[1] Department of Electrical Engineering, Sejong University, 209 Neungdong-ro, Gwangjin-gu, 05006, Seoul, Korea.

[2] School of electronics Engineering, Chungbuk National University, Cheongju 28644, South Korea.

[3] Department of Convergence Engineering for Intelligent Drone, Sejong University, 05006, Seoul, Korea.

* Correspondence: mfk@sejong.ac.kr (M.F.K); and deokkeekim@sejong.ac.kr (D-k.K.)

† These authors contribute equally.

Atomic force microscopy of $MoTe_2$.

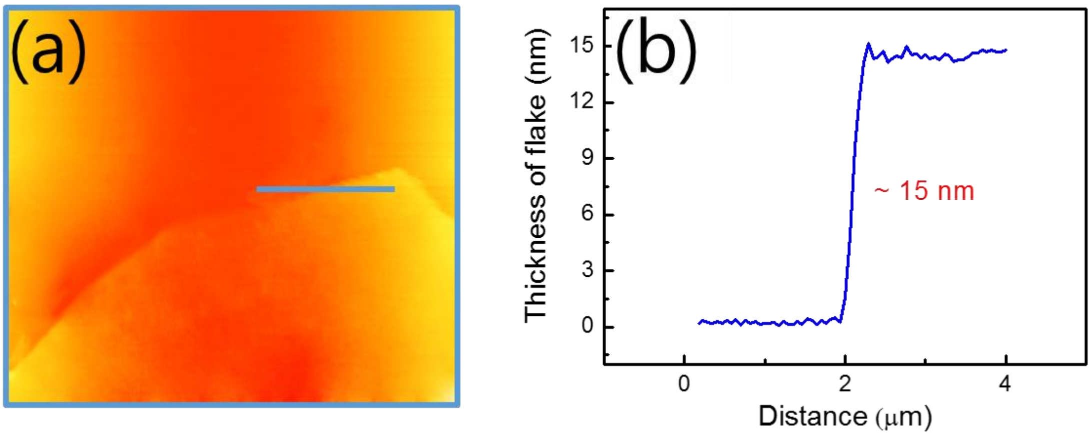

Figure S1. (**a**) The atomic force microscopic image of the few layer $MoTe_2$. (**b**) The height profile of $MoTe_2$ flake along the line scan.

Gate dependent electrical and post-synaptic current measurements of $MoTe_2$.

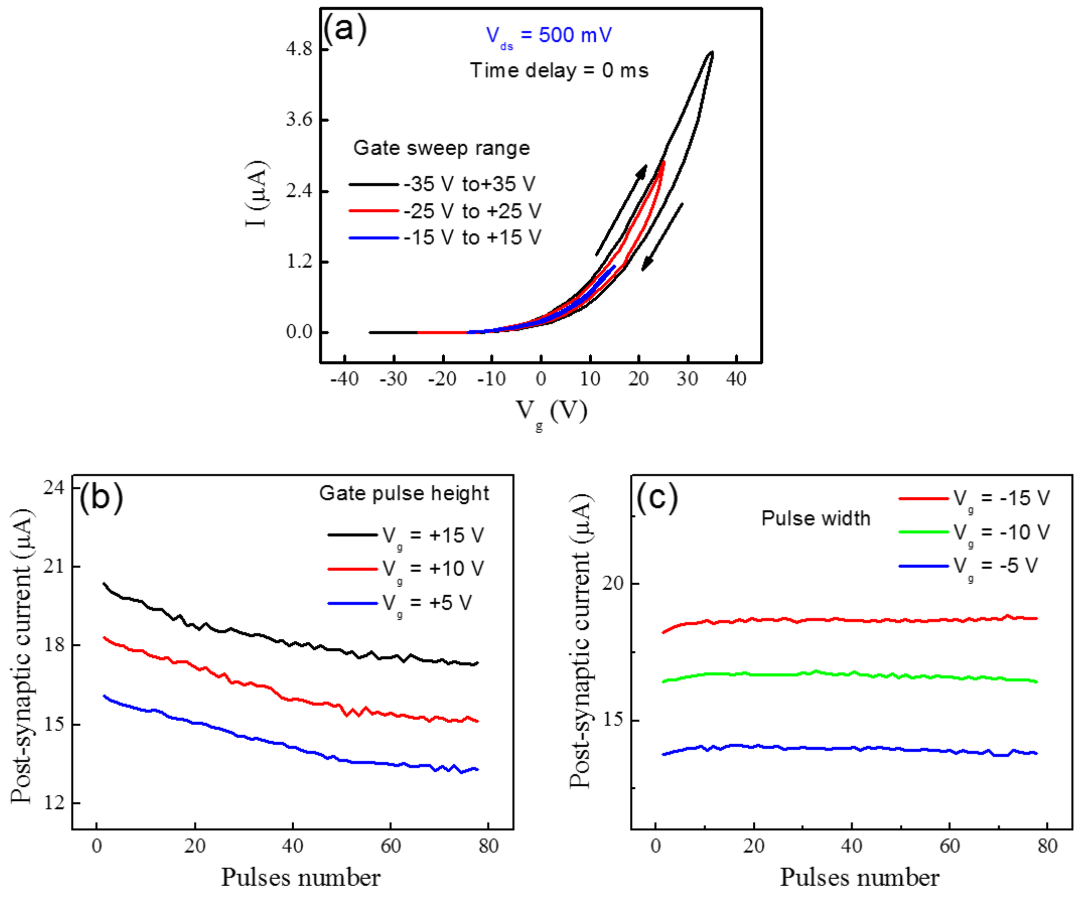

Figure S2. (**a**) Transfer characteristics of the $MoTe_2$ FET with hysteresis loops at different back gate sweep ranges. (**b**) The PSC vs. pulse number of the $MoTe_2$ FET by applying positive gate voltage with increasing height pulses. (**c**) The PSC vs. pulse number of the $MoTe_2$ FET by applying negative gate voltage with increasing height pulses.
